# Revisiting corporate universities: Strategic choices shaping performance in telecom

**DOI:** 10.1016/j.heliyon.2024.e34314

**Published:** 2024-07-09

**Authors:** Elena Shakina, Ángel Barajas, Patricio Sánchez-Fernández

**Affiliations:** a GEN- Governance and Economics Research Network, Universidad de Vigo, Campus de Ourense, As Lagoas, 32004, Ourense, Spain; b International Laboratory of Intangible-driven Economy, HSE University; c Departamento de Economía Financiera y Contabilidad, Universidad de Vigo, Campus de Ourense, As Lagoas, 32004, Ourense, Spain; d GEN- Governance and Economics Research Network, Universidad de Vigo, Campus de Ourense, As Lagoas, 32004, Ourense, Spain

**Keywords:** Corporate university, Quasi-experiment, Firm performance, Propensity score matching, Synthetic control group

## Abstract

Corporate universities (CUs) have evolved significantly over the past century, yet empirical studies examining their impact on corporate performance are notably sparse. This study addresses this gap by applying two alternative quasi-experimental designs - Propensity Score Matching (PSM) and Synthetic Control Group (SCG), to assess CU's strategic value. By examining 18 listed European telecom companies and 80 Spanish corporations across various industries during the 2004 to 2018 time span, this paper presents a comparative analysis of CU implementation effects within different corporate environments. Our findings indicate that CU establishment is a strategic choice for better corporate performance, identity, and strategy rather than an isolated educational investment. The results offer a better understanding of CU's strategic role, especially in sectors characterized by rapid technological change and the need for strategic human capital development. This study contributes to both empirical knowledge and methodology, proposing innovative ways to explore the effects of strategic decisions like CUs on corporate performance, and enhancing the dialogue between strategic human resource management and corporate investors in the scholarly and managerial domains.

## 1 Introduction

This paper comes to find evidence of whether setting up a corporate university (CU) can be a reasonable strategic choice for a company. The phenomenon of CU first appeared over a century ago and has undergone significant transformation since then. A contemporary CU overruns a very significant part of social responsibility programs, HR practices or even represents an independent business unit of large corporations. But does a CU always accomplish its original objectives? And do companies while establishing CUs pursue manifold corporate objectives? There is still little empirical evidence to answer these questions.

Rather few research attempts like those by Refs. [[1], [2], [3], [4], [5]] have been undertaken so far. If looking at the landscape of relevant papers in the Scopus database there are no more than 120 contributions throughout the entire publication history and only not more than 20 % of those papers seek to answer the research question on the CU-performance relationship. This paradox rather refers to the complexity of testing this phenomenon than its irrelevance. One of the main issues raised in academic research addresses the rarity and incomparability of different CU cases [6]. For that reason, the majority of CU-related studies like those by Refs. [7,8] draw single or few cases of evidence. That leads to a fairly limited generalizability of the findings and makes CUs hard to analyze in light of their impact on corporate performance lacking counterfactual conditions.

Furthermore, the conventional understanding of CUs proposes them as a significant investment, which cannot be a standalone strategic decision. CUs are intrinsically linked with other strategic corporate domains and cannot be isolated from the rest of the company's activities. For instance, the integration of CUs with talent management, leadership development, and organizational learning initiatives demonstrates how deeply CUs are embedded within the strategic fabric of a corporation [[8], [9], [10]]. This interconnectedness suggests that the impact of CUs on corporate performance is mediated by various factors, such as the alignment with business goals, the support from top management, and the adaptability to changing market conditions.

Moreover, CUs typically do not deliver immediate effects. Their benefits are often realized over the long term as they contribute to the development of human capital, foster innovation, and enhance organizational capabilities [3]. This delayed impact makes it tricky and speculative to determine the exact lag between CU launch and measurable improvements in performance. The time required for a CU to produce tangible results can vary significantly based on the industry context, the specific strategic objectives it aims to support, and the effectiveness of its implementation.

All these factors together make CU-driven corporate performance a challenging puzzle for empirical tests. The complex nature of CUs, their integration with other strategic domains, and the delayed realization of their benefits complicate the assessment of their true impact on corporate performance. Consequently, empirical endeavours need to account for these complexities by adopting comprehensive research designs that capture the long-term and indirect effects of CUs within the broader strategic landscape of the organization.

Thus, despite CUs’ strategic relevance and evolving nature which is observed in recent years, empirical research exploring their impact on corporate performance remains surprisingly sparse. This gap is reflected in the limited number of comparative studies that probe into the relationship between CU initiatives and corporate outcomes. Although over a century old and increasingly integral within corporate strategy, CUs are not often the focus of rigorous academic inquiry, with only a minor fraction of the existing literature. A corporate decision to establish CU cannot be considered a random natural experiment. Apparently, these decisions are endogenous and largely depend on previous performance, corporate identity and strategy, and a set of unobservable factors. That critically limits available experimental methods. In response to this evident research gap, this paper suggests revisiting the CU phenomenon and introduces two alternative quasi-experimental designs utilizing Propensity Score Matching (PSM) and Synthetic Control Group (SCG) methods to tackle the methodological challenges presented by CU cases. These techniques offer a novel approach to compare the effects of CU implementation across different corporate environments, aiming to provide more generalized and robust findings than those typically obtained from conventional case studies. A principal difference between the two quasi-experimental procedures is attributed to the way the counterfactual conditions are built. One of the advantages of the research design, as implemented in this study, refers to the fact that having a limited number of observed cases of CUs, we can run a reasonably precise comparison. Both PSM and SCG procedures do not require a big number of observations for robust inference.

By focusing on the telecom industry in Europe, this study not only contributes to filling the empirical void but also enhances our understanding of CU's strategic value in a specific sector known for rapid technological advancements and strategic human capital development.

For our experiment, we use data from 18 listed European telecom companies observed from 2004 till 2018 and 80 largest Spanish corporations across all industries. The panel data contains 270 telecom company-year observations and 1200 company-year observations in Spain. The data has been collected by the International Laboratory of Intangible-driven Economy at the HSE University in several waives. The financial data has been retrieved from Bureau van Dijk (Amadeus), Bloomberg and Thomson Reuters. The non-financial information including information on CU, knowledge management implementation and qualification of boards of directors has been obtained from annual corporate reports and the official website of the companies.

This study contributes primarily to the empirical knowledge of CU effectiveness advancing the understanding of CU-performance paradigm through its innovative dual-model empirical approach. Finding a compromise between comparative and traditional single-case studies, our research employs two alternative quasi-experiments, offering a more granular evidence-based analysis of CUs' impact. This dual-modality approach not only overcomes the limitations of rarity and incomparability of CUs but also provides a more robust and generalizable understanding of their strategic implications. Our study reveals that the impact of CUs on firm performance is highly heterogeneous, indicating that it is not possible to make a definitive claim about their universal value to firms. However, in specific instances, the effectiveness of CUs can be assessed using the SCG technique. This approach, as demonstrated in our analysis of Telefonica, shows that the CU was beneficial for enhancing firm productivity and had a marginally positive effect on its attractiveness to corporate investors, as evidenced by market value metrics.

The paper is organized into five sections. Whereas this study seeks to contribute empirically and methodologically, there is a detailed description of the existing empirical papers on CU-driven performance. The selected European CU cases enable diving into the study context and justify the measurements and quasi-experimental conditions. Moreover, a special section on methodology suggests a comparison of two quasi-experimental alternatives as proposed in this study. The paper is concluded with the results’ description and discussion.

## 2 CU and performance: an academic and expert discussion

### 2.1 What is a contemporary CU?

CUs have become a prominent feature of contemporary corporate strategic choice. A CU can be defined as an organization's internal educational system that provides training, development, and learning opportunities to its employees [11]. The concept of CUs has been around for over a century, but it has undergone significant transformations since its inception [8,12]. The earliest forms of CUs were created to provide training and development opportunities to employees, but over time, their scope and function have expanded. Today, CUs are seen as an integral part of corporate strategy and have taken on a much broader role, including the development of corporate culture, the promotion of employee engagement and loyalty, and the improvement of the company's reputation [13,14].

Although CUs are not designed to substitute traditional universities, they present according to Ref. [15] a useful strategic choice for corporations seeking to enhance their workforce's professional development and maintain competitiveness amidst an ever-evolving business climate. As strategic assets, CUs allow companies to tailor educational and training programs to meet specific organizational needs, bridging the gap between academic knowledge and practical industry skills. This alignment ensures that employees are equipped with the latest competencies required to drive innovation and achieve strategic objectives [16].

By investing in CUs, corporations can foster a culture of continuous learning and development, which is essential for adapting to rapid technological advancements and shifting market demands [17]. Unlike traditional universities, CUs can quickly respond to these changes, providing customized training that addresses current challenges and future trends in the industry. This agility makes CUs a vital component of a company's strategic planning, as they can proactively prepare the workforce for emerging opportunities and threats.

Moreover, as [10,18] assert CUs support strategic initiatives such as leadership development, succession planning, and knowledge management. They play a crucial role in identifying and nurturing talent within the organization, ensuring that future leaders are well-prepared to take on critical roles. This internal development pathway is a strategic move to retain top talent and reduce reliance on external hires, which can be costly and time-consuming.

In addition to enhancing individual capabilities, the study by Ref. [1] suggest the CUs contribute to organizational performance by fostering collaboration and knowledge sharing across departments and geographies. They create a unified learning environment where best practices and innovative ideas can be exchanged, leading to improved efficiency and effectiveness in achieving strategic goals. Furthermore, the presence of a CU can enhance a corporation's reputation as an employer of choice according to Ref. [15], attracting high-caliber candidates who value professional growth opportunities. This strategic advantage in talent acquisition further strengthens the company's competitive position in the market.

[19] literature review suggests that corporate universities may help alleviate intersectoral knowledge hiding by enabling employees to share the latest industry knowledge and expertise from leading professionals.

In the study by Ref. [2], CU is positioned as a measure for a company to stay ahead of the curve by providing employees with the latest industry knowledge, trends, and innovations. This can help the company to become a leader and a knowledge disseminator [18]. Moreover, it allows one to be more innovative, adaptable, and proactive, which can lead to increased competitiveness in the market [9].

CUs, according to Ref. [2], can also help to improve employee retention and reduce the cost of recruiting and training new employees. This can lead to increased organizational stability, consistency and efficiency, which can be particularly important for shareholders. The authors also explore how CUs can foster organizational ambidexterity, focusing on their role in facilitating the deployment of resources necessary for balancing exploration and exploitation—key for firms operating in rapidly changing markets. Their study of ZTE University exemplifies how CUs can be dynamically linked to the development of capabilities that allow for strategic flexibility. Further [1], delve into the intellectual capital within CUs, emphasizing the role of knowledge networks in shaping this capital. Their research highlights how CUs contribute to synergizing teacher network capital, knowledge process capital, and knowledge, enhancing the organizational knowledge network essential for coordination and innovation within corporate settings.

Furthermore, CUs can also help to develop a strong company culture and a positive reputation, which can help to attract and retain talented employees and customers and increase the company's brand value. That, in turn, exhibits a set of positive signals for corporate investors and is seen one of the relevant corporate social innovation tools according to Ref. [20].

On the historical and cultural front [21], provides insight into how global business education models, particularly the American model, have influenced corporate universities. His analysis of the Helsinki School of Economics/Aalto University School of Business over several decades illustrates the impact of increased corporatization on educational strategies and outcomes, reflecting broader trends in global business education. Following this idea [22], investigate how CUs support lifelong learning within enterprises. They identify how CUs generate and share strategic and business knowledge, optimizing governance and transforming cultural knowledge, thus playing a crucial role in fostering continuous learning and adaptation among employees in an era of rapid change [23]. discuss how managerial ideologies, particularly during crises such as COVID-19, have accelerated the corporatization within universities. Their study points out the transformation in academic roles and university operations, underlining the shift towards revenue-centric models and its impact on academic identity and governance.

There is then a class of literature discussing the CUs' role in different national contexts [24]. set CU initiatives within the national context of China, investigating the case of Haier CU's critical role in knowledge management through three interlinked activities: operational knowledge transfer, networking, and scientific and technological activities. These activities form a dynamic “figure of eight” cycle with networking at the core, facilitating integration and enhancing the effectiveness of knowledge management in Chinese CUs. Later [25], analyze different models of CUs in South Korea, using a holistic framework to assess their sustainability. Their findings suggest that a balanced approach, incorporating various types of CUs, can lead to sustainable outcomes by aligning educational strategies with organizational needs.

Despite the diverse empirical endeavors on the CU phenomenon for an organization, a substantial gap remains in understanding the impact of CUs on organizational performance. Meanwhile, there is a pressing need for more empirical studies using robust quantitative methods to measure the performance outcomes of CU initiatives. Additionally, research should explore the long-term impacts of CUs on organizational strategy and performance, particularly how they adapt to and integrate with rapidly evolving corporate environments. The next section underscores the complex and multifaceted roles of CUs in modern corporations, highlighting the need for further research to fully understand their strategic implications and contributions to corporate success.

### 2.2 CU-driven performance

The relationship between CUs and corporate performance is a complex issue that has attracted the attention of researchers and practitioners alike. While the potential benefits of CUs, such as improved employee performance, innovation, and organizational learning, are well-recognized, empirical evidence on their direct impact on corporate performance remains limited. This gap in the literature underscores the need for further research to better understand the conditions under which CUs can effectively contribute to a firm's strategic objectives.

If CUs are considered as one of the strategic resources of firms, previous research explicitly or implicitly refers to the resource-based view (RBV) by Ref. [26]. The RBV posits that a firm's competitive advantage is derived from its ability to acquire, develop, and effectively deploy valuable, rare, inimitable, and non-substitutable resources. Within this framework which is mentioned by Refs. [4,27,28], CUs can be seen as a strategic asset that contributes to building and sustaining a firm's competitive advantage by enhancing the skills and capabilities of its workforce. Moreover, the academic literature [15,22,29] suggests that the impact of CUs on corporate performance may be contingent on several factors, including the alignment of CU programs with the firm's strategic goals, the level of support from top management, and the integration of CU initiatives with other strategic HR practices. Therefore, adopting a resource-based perspective provides a valuable lens through which to examine the strategic role of CUs and their potential to enhance corporate performance.

However, the impact of CUs on corporate performance is still a matter of debate among researchers and practitioners [2,30,31]. There is not much proof to back up the idea that a CU always accomplishes its desired goals and that creating one will result in better overall performance for a company.

The paper by Ref. [4] evidenced that CUs can influence a firm market value added and convince its shareholders by improving the overall performance and competitiveness of the company. Meanwhile, by providing employees with the skills and knowledge necessary to perform their job duties more effectively, CUs can help the company increase its productivity, efficiency, and quality, which can lead to improved financial performance [2,24]. Of note, the latter study focuses on the strategic role of CUs in fostering organizational ambidexterity within technology-based companies. The authors argue that CUs can effectively deploy resources and capabilities necessary for balancing exploration and exploitation, which are critical for maintaining competitiveness in rapidly changing markets. This suggests that CUs could potentially enhance company performance by fostering strategic flexibility and innovation. However, the study is based on a single case, which might limit the generalizability of its findings across different organizational contexts or industries.

In a similar vein [25], explore the sustainable development of CUs and discuss how these institutions can address specific organizational needs through tailored educational programs. Their findings suggest that aligning CU activities with corporate strategies can significantly enhance organizational capabilities and indirectly boost company performance. However, the problem arises in ensuring that these educational initiatives are not only well-integrated but also continuously adapted to evolving corporate strategies and market conditions, which can be a significant challenge for management.

The establishment of a CU is a significant investment for a company, and it is not a standalone strategic decision. CUs are bound with strategic human resource management and social responsibility agendas, and likely, they cannot be isolated from the rest of the company's activities. For instance Ref. [1], considering CUs as a part of corporate strategic intellectual resources along with [4,32], emphasizing how knowledge networks can enhance a company's innovation capacity and competitive edge. These investigations highlight the potential of CUs to contribute to corporate performance by facilitating sophisticated knowledge management practices. Nonetheless, the problematic aspect here involves the creation and maintenance of effective knowledge networks that can truly capture and leverage intellectual capital in ways that are measurable and impactful on performance. In addition, the effect of a CU on a company's performance is not immediate and it can be difficult to identify the time lag between when a CU is established and when performance is improved.

Thus, one of the main issues raised in academic research on CUs is the rarity and incomparability of different CU cases. This makes it challenging to analyze the impact of CUs on corporate performance in a rigorous and systematic manner.

Despite these challenges, there is evidence to suggest that CUs can have a positive impact on corporate performance. A study by Ref. [3] found that CUs can lead to improved employee satisfaction and increased innovation, both of which are critical factors in the success of any organization [8,33]. also found that CUs can lead to improved employee motivation and engagement, which can, in turn, lead to improved corporate performance.

Additionally [5], found that CUs can play a critical role in the development of a company's corporate culture and the promotion of employee engagement and loyalty. This, in turn, can lead to improved corporate performance and increased competitiveness in the market.

To conclude, the academic discourse primarily focuses on the mechanisms through which CU impact corporate performance, rather than debating the existence of this relationship, which is widely accepted by both scholars and experts.

Given the complexities involved as discussed by Ref. [4], future research should aim to explore the mechanisms through which CUs contribute to corporate performance, considering both direct and indirect effects. Longitudinal studies and in-depth case analyses could provide richer insights into how CUs, as strategic resources, influence firm outcomes over time. Additionally, comparative studies across different industries and organizational contexts could help identify best practices and critical success factors for leveraging CUs as part of a firm's strategic resource portfolio.

Thus, our study aims to challenge the assumption of the straightforward CU-performance link by considering the possibility that it may not be as apparent as previously thought. In the following section, we will outline the methodological approach employed in this paper and provide the econometric strategy used in the two studies.

## 3 Research program: hypothesis development and research design

The research program is designed to bridge the existing gap in the empirical literature and suggest alternative methodological solutions for the CU-perfromance puzzle. The strategic role of CUs and their translation into corporate performance, are differentiated into two specific dimensions: operational effectiveness and CUs’ attractiveness to corporate investors. These two critical dimensions are grounded in contemporary scholarly discourse, as delineated in the preceding section. The following hypotheses are formulated to guide the empirical exploration of our study.

Hypothesis 1 The strategic implementation of CU enhances operational effectiveness, reflected in improved firm productivity.

The rationale of this hypothesis suggests that corporate universities serve as a strategic tool to boost operational effectiveness by providing targeted employee development programs, which in turn lead to increased firm productivity. The effect is expected to be observed universally, yet the magnitude may vary in response to the distinctive features of a firm.

Hypothesis 2 The presence of a CU elevates a firm's attractiveness to corporate investors, as evidenced by an increase in market value added (MVA).

The rationale behind this hypothesis challenges whether CUs are posited to be a signal of a firm's long-term commitment to human capital development, which bolsters investor confidence and, consequently, the firm's market value. This attractiveness, however, is presumed to be influenced by a comparative base of alternative investment strategies.

In our research, we examine and compare the results of two studies, Study 1 and Study 2, that are based on different quasi-experimental designs. Both Study 1 and Study 2 were conducted to explore the impact of CU on corporate performance. However, they differ in the methods they employ to control extraneous variables and establish causality. Study 1 used a non-equivalent control group design, while Study 2 utilized a pre-post treatment design.

Study 1 seeks to establish the average treatment effect of the CUs fitting the treatment and the control groups on the base of non-random assignment of CU launch. Among those companies, 5 have a CU established either during or before the observation period 2004–2018. Study 2 implies testing one of the five CU cases. The case of Telefonica's CU has been chosen because its launch took place within the analysed period in 2009.

By comparing the results of these two studies, we aim to set a CU treatment effect on performance in two different dimensions: operational effectiveness and attractiveness for corporate investors and gain better understanding strengths and limitations of different quasi-experimental designs. Of note, this two-pronged approach allows shedding some light on the robustness of the findings. Furthermore, the comparison provides valuable insights into the impact of design choices on the generalizability of research results and the validity of conclusions. Through this comparison, we hope to contribute to the ongoing debate on the use of quasi-experimental designs to test the CU-performance relationship. Before discussing the methodological issues and drawing the results, the institutional context of Study 1 and Study 2 is introduced in the following subsections.

### 3.1 Study 1: telecom in Europe and CU

We chose the telecom industry due to its distinctive characteristics that make it a good context for exploring the strategic value of CUs. The telecom sector is renowned for its rapid pace of technological innovation, which necessitates a constant update of skills and knowledge. The requirement for specialized training to keep pace with technological advancements is more pronounced in telecom than in many other sectors. This sector relies heavily on cutting-edge knowledge and the application of such knowledge, which can significantly influence operational effectiveness and competitive advantage. Moreover, the telecom industry has a substantial impact on a wide array of economic activities, and its performance is closely linked to the effectiveness of its human resources. It is in this high-stakes environment that CUs can play a crucial role in ensuring a workforce that is agile, well-informed, and equipped with the latest skills. The potential for CUs to contribute to corporate performance, therefore, is arguably more tangible and immediate in telecom than in industries with less reliance on rapid innovation cycles.

In Europe, some of the largest telecom companies include Vodafone, Deutsche Telekom, and Telefonica (see Table 1).

**Table 1:** Largest European telecom companies (treatment/control groups).

	Company name	Treatment/CONTROL Group	
1	VODAFONE GROUP PUBLIC LIMITED COMPANY		1
2	TELEFONICA, SA	1	
3	DEUTSCHE TELEKOM AG	1	
4	FRANCE TELECOM	1	
5	BT GROUP PLC		1
6	COBHAM PLC		1
7	JAZZTEL P.L.C.		1
8	TT ELECTRONICS PLC		1
9	TELECOM PLUS PLC		1
10	KCOM GROUP PUBLIC LIMITED COMPANY		1
11	SPIRENT COMMUNICATIONS PLC		1
12	MAINTEL HOLDINGS PLC		1
13	BD MULTIMEDIA	1	
14	BUDGET TELECOM	1	
15	2ERGO GROUP PLC		1
16	INDEX MULTIMEDIA	1	
17	IPPLUS PLC		1

These companies offer a wide range of services, including mobile and fixed-line telephony, broadband and internet services, as well as pay-tv and multimedia services. They have invested heavily in the development of cutting-edge technologies, such as 5G networks, and are at the forefront of the digital transformation of the continent.

To stay ahead of the curve and meet the ever-changing demands of their customers, some telecom companies in Europe have established CUs. In our sample, those corporations like Telefonica, Deutsche Telekom, BD Multimedia, Budget Telecom, and Index Multimedia, declare CUs as a part of their HR and CSR programs. According to the information disclosed in the annual corporate reports and on the official websites, these institutions provide training and development opportunities for employees, enabling them to acquire new skills and knowledge in areas such as technology, innovation, and leadership.

For example, Deutsche Telekom offers a variety of programs and courses in areas such as technology, marketing, and management. Similarly, BD Multimedia has created its CU, a learning and development centre that provides training programs and workshops to employees. These CUs not only help to develop the skills and capabilities of employees but also contribute to the overall success and competitiveness of the companies. However, there are big European leaders in telecom which have not ever decided on a CU launch. One of the sound examples is Vodafone Group plc. Vodafone, like many other telecom corporations, has not seen CU as a strategic choice. Vodafone may have determined that the cost outweighs the potential benefits of CU or have decided to focus on other strategic priorities. Thus, it is worth noting that the decision not to launch CU does not necessarily mean that a company cannot afford this investment because a perceptible number of big telecom leaders do not consider CU among their priorities. Said that, we may suggest that if similar companies with PSM selection enter a quasi-experiment, the average causal effect of CU on performance can be identified.

### 3.2 Study 2: telefonica CU case

Telefonica is a Spanish telecommunications company founded in 1924. It is one of the largest telecom companies in the world, operating in Europe, Asia, and America. The company provides a wide range of services including fixed and mobile telephony, broadband and multimedia services, as well as technology solutions for businesses and governments. Telefonica's operations in Europe are centred in Spain, where it is the largest provider of telecommunication services. It also operates in other countries such as the UK, Germany, and Portugal, among others. In Asia, the company has a strong presence in countries like China and India, and in the Americas, it has operations in countries like Brazil, Chile, and Peru.

CU is the educational arm of Telefonica. It was established in 2009 to provide high-quality training and development opportunities for employees of the company.^1^ According to the official information retrieved from Telefonica's annual reports and website, the goal of the university is to help employees develop the skills and knowledge they need to succeed in their careers and contribute to the success of the company.

Telefonica CU offers a wide range of programs and courses, including technical training, leadership development, and digital transformation. The university also provides opportunities for employees to participate in international exchanges and programs that allow them to gain new perspectives and build their global networks. The selection of Telefonica for Study 2 was driven by several factors. Firstly, as one of the largest telecommunications companies in the world, Telefonica is a market leader in Spain, providing a rich case study of a CU's impact within a major player in the industry. Its influence on the Spanish market and beyond allows for a meaningful analysis of strategic educational initiatives on a large scale. Moreover, Telefonica has been at the forefront of implementing innovative corporate educational strategies. This positions the company as a compelling subject for evaluating the efficacy and strategic value of CUs. Secondly, Telefonica offers a sufficiently long span of data both before and after the establishment of their corporate university. This duration is essential to effectively implement the SCG method, as it allows for the analysis of the CU's impact over an extended period, accounting for trends and cycles that affect corporate performance. The availability of data over these periods ensures that we can observe the full effect of the CU launch, encompassing any lag effects and long-term changes in productivity and market value added. Lastly, while Telefonica is a specific case, it presents a scenario from which broader implications for the telecom sector, both within and outside of Spain, can be drawn. Insights gained from this study may be applicable to other firms with similar structures and strategic objectives.

Currently, we can study the Telefonica CU case for at least 10 years and evaluate its CU-driven corporate performance by comparing it to quasi-experimental counterfactual scenarios. In Study 2, we will create synthetic control groups in two settings - other European telecom corporations and large listed Spanish companies - to determine the impact of the CU launch. We will select the control group in two ways to evaluate Telefonica CU's relative performance from two perspectives. That will help us identify the conditional effects of th CU launch under different benchmarking.

### 3.3 Methodology and measurements

Our investigation employs two alternative quasi-experimental research strategies: propensity score matching (PSM) combined with an Ordinary Least Squares (OLS) estimator, and Synthetic Control Group (SCG) analysis. PSM is a statistical technique applied for Study 1 and seeks for CU effect by fitting treatment and control groups based on their general similarities and testing whether a treatment – CU establishment in the case of our study – makes a difference in the residual performance which is not explained by other factors. SCG utilized for Study 2 is not a statistical but rather an optimization procedure that enables the comparison of a treated unit with the synthetic control composed of relevant characteristics of counterfactuals in the pretreatment and posttreatment periods – before and after CU was launched. Being close in their nature, these two procedures are fundamentally different in the way they demonstrate the effect of CUs on performance. PSM reveals a cross-sectional intertemporal effect for a group of treated companies while SCG picks the individual effect of each particular treated company at every post-treatment point in time.

#### 3.3.1 Propensity score matching

PSM is a statistical method that was proposed by Ref. [34] as an alternative to evaluating the average treatment effect for settings when observations are substantially heterogenous and treatment is questionably exogenous. Now is widely used in quasi-experimental design to account for the effect of treatment. In this paper, PSM is implemented to reveal the average treatment effect of CU establishment on an outcome variable - corporate performance. PSM is used to control for observed and unobserved confounding variables that may affect the relationship between the treatment and outcome variables. Hence, the purpose of PSM is to balance the distribution of observed covariates between treatment and control groups, so that any differences in outcomes between the groups can be attributed to the treatment and not to differences in the distribution of covariates.

For the purpose of this paper, PSM is used to study the performance effect of CU. The first step in the PSM process is to estimate the propensity score for each observation in the sample. The propensity score is a predicted probability of receiving the treatment (i.e., establishing a CU) based on a set of observable covariates that may affect the treatment decision. The covariates are chosen based on prior knowledge and research on the relationship between CU establishment and performance, as well as factors that may confound the relationship.

After estimating the propensity scores, the next step is to match each treated observation (i.e., companies with established CUs) with one or more control observations (i.e., companies without CUs) based on the nearest neighbour algorithm or other matching methods. The objective of matching is to create a control group that is similar to the treatment group in terms of the distribution of observed covariates [35]. This helps to ensure that any differences in outcomes between the groups prior to CU treatment are not due to differences in the distribution of covariates.

Once the control and treatment groups are created, the OLS (Ordinary Least Squares) estimator is used to estimate the average treatment effect of CU establishment on corporate performance. The OLS estimator provides a linear regression model that estimates the effect of CU establishment on performance, controlling for the covariates used in the propensity score estimation. The OLS estimator can provide a point estimate of the treatment effect, as well as confidence intervals and hypothesis tests that can be used to assess the statistical significance of the treatment effect.

The technical explication of the PSM coupled with OLS is represented algebraically as follows:

Propensity Score Estimation:

Let $X_{i}$ represent a vector of observable characteristics for unit.

The propensity score $p\left(X_{i}\right)$ is the probability of receiving the treatment given these characteristics and is estimated typically using a logistic regression:

$$p\left(X_{i}\right)=\frac{e^{\left(X_{i}^{\prime }\beta \right)}}{1+e^{\left(X_{i}^{\prime }\beta \right)}}$$

where.

e is the base of the natural logarithm,

β is a vector of parameters to be estimated, and is the transpose of $X_{i}$.

Units in the treatment group *T* are matched to units in the control group *C* based on their propensity scores.

Outcome Analysis with OLS:

$$Y_{i}=\beta_{o}+\delta T_{i}+\gamma D_{i}+CV+\epsilon_{i}$$

where.

$Y_{i}$ is an outcome variable (productivity and MVA),

$T_{i}$ – treatment of CU,

$D_{i}$ – distance between matched groups as an output of PSM,

CV – vector of control variables,

$\epsilon_{i}$ – error term.

The advantage of using PSM with OLS is that it allows for control of observed and unobserved confounding variables that may affect the relationship between the treatment and outcome variables. This is important in this study, as the decision to establish CU is not a random process, and may be influenced by factors such as previous performance, corporate identity, and strategy. By controlling for these confounding variables, PSM with OLS can provide a more accurate estimate of the effect of the treatment on the outcome variable.

#### 3.3.2 Synthetic control group method

The SCG method as a quasi-experimental design is used in our paper to estimate the effect of a CU treatment for one of the telecom companies. SCG is a relatively new method proposed by Refs. [36,37]. This method is often used in the absence of a randomized control group or when a randomized control group is not feasible like in CU cases. The SCG method involves creating a synthetic control group that mimics the pre-treatment characteristics of the treated unit as closely as possible. The synthetic control group is created by combining the characteristics of multiple control units that are similar to the treated unit in the pre-treatment period.

In the SCG method, the treated unit's post-treatment outcomes are compared to the outcomes of the synthetic control group to estimate the effect of the intervention. The key assumption of the SCG method is that the synthetic control group accurately represents the counterfactual outcome of the treated unit if the intervention had not been implemented.

The synthetic control group is created using a two-step process. First, the relevant characteristics of the control units are identified and measured in the pre-treatment period (before CU was launched in our study). Second, the weights for each control unit are determined such that the synthetic control group accurately represents the treated unit's characteristics in the pre-treatment period.

The SCG method can be considered as an optimization problem where the objective is to minimize the distance between the treated unit's characteristics in the pre-treatment period and the characteristics of the synthetic control. The optimization problem is solved using linear programming algorithms. The weights assigned to each control unit represent the relative importance of each unit in creating the synthetic control.

The SCG approach is designed to estimate the causal effect of an intervention when a randomized experiment is not feasible. It relies on a combination of pre-treatment characteristics to form a synthetic control, against which the post-treatment outcome of the treated unit is compared to estimate the treatment effect.

The Synthetic Control Group (SCG) method can be technically expressed as follows.

1. Creation of Synthetic Control:

Let $Y_{1}^{T}$ denote the post-treatment outcome for the treated unit, and $Y_{j}^{C}$ for the control units. The synthetic control is constructed by selecting a weighted combination of control units that approximates the characteristics of the treated unit before the intervention. This can be represented as:

$$Y_{i}^{synthetic}=\sum_{j=2,i=1}^{J,N-1}W_{j}\times Y_{ji}^{C}$$

Where: $Y_{i}^{\text{synthetic}}$ - is the outcome for the synthetic control in period i, N – treatment period.

$W_{j}$- are the weights assigned to each control unit such that $\sum W_{j}=1$ and $W_{j}\geq 0\text{,}$ and $Y_{j}^{C}$ is the outcome for control j unit in the pre-treatment period from 1 to N−1 to minimize the difference between $Y_{1}^{T}$ and $Y_{i}^{\text{synthetic}}$ in the pre-treatment period.

2. Estimation of Treatment Effect: The treatment effect (TE) for the treated unit after the intervention is the difference between the actual outcome and the outcome for the synthetic control:

$${\text{TE}}_{i}=Y_{i}^{T}-Y_{i}^{\text{synthetic}},i\;\text{from}\;N\;\text{to}\;\text{the}\;\text{last}\;\text{period}\;\text{of}\;\;\text{observations.}$$

This difference represents the estimated effect of the treatment on the treated unit.

The advantage of the SCG method over other quasi-experimental designs is its ability to estimate the individual effect of each treated unit in every post-intervention period. This makes the SCG method well-suited for studies with a limited number of treated units. Additionally, the SCG method does not require large numbers of observations for robust inference, making it useful in cases where there is limited data available.

### 3.4 Measurement of CU-driven performance and covariates

The impact of CU on corporate performance is a topic of debate in the literature as has been demonstrated in the previous sections. To contribute to this discussion, we focus on the measurement of CU-driven performance and its relationship with a set of covariates that may condition this relationship.

Measuring CU-driven performance is a complex task, given the multifaceted nature of the construct and the diverse approaches to operationalizing it. In this study, we adopt a comprehensive view of CU-driven performance that encompasses various dimensions, such as internal productivity, and outcomes for investors. We use a combination of objective financial indicators to capture the different aspects of CU impact. To enhance the validity and reliability of the measures, we suggest the overview of phenomena to be captured by different metrics as given in the previous studies (see Table 2).

**Table 2:** Measurements employed for the analysis.

Phenomenon to be explored	Measurement	Previous studies
Performance		
	Productivity^2^	[38,39]
	Market value added (MVA)	[4]
Covariates		
	Economic value added (EVA)	[4]
	Firm's age	[4,40]
	Book value	[39]
	Capital expenses	[4]
	Return on invested capital (ROIC)	[4]
	Board of Directors qualification^3^	[3]
	Employee cost share^4^	[27,28,40]
	Foreign capital^5^	[3,28,43]
	Location in big city^6^	[4]
	Knowledge management system adopted^7^	[24,31]

The measurements proposed in Table 2 are used for both Study 1 and Study 2. The following subsection gives the description of the variables.

### 3.5 Data for studies 1 and 2

Descriptive statistics provide a useful way to summarize and analyze dataset, allowing to gain insights into key benchmarks taken for the quasi-experiment in Study 1 and Study 2. In this study, we examine descriptive statistics of telecom companies, with a focus on key performance indicators and covariates as given in Table 1. Table 3, Table 4 demonstrate two sub-settings for telecom industry (Study 1 and Study 2) and all the largest corporations in Spain (Study 2). Telecom companies have experienced significant growth in recent years, driven by advances in technology, increased demand for data and voice services, and changes in consumer behavior.

**Table 3:** Descriptive statistics of telecom companies.

Statistic	N	Mean	St. Dev.	Min	Pctl(25)	Pctl(75)	Max
Productivity	270	−0.01	0.12	−0.74	−0.01	0.04	0.17
Market value added (MVA)	270	−691.17	13,632.95	−59,532.90	−3.90	361.53	45,647.92
Economic value added (EVA)	255	−1,075.79	3,498.30	−31,124.04	−274.00	4.08	5,397.20
Firm's age	270	34.33	34.36	1	13	30.8	129
Book value	270	30,440.44	53,442.46	0.31	15.99	34,891.35	194,455.90
Capital expenses	270	2,638.81	5,936.23	−11,182.36	0.09	1,872.21	41,428.00
Return on invested capital (ROIC)	270	0.06	0.17	−0.49	0.02	0.11	0.92
Board of directors' qualification (BoDQ)	270	1.06	0.67	0	1	2	2
Employee cost share	215	0.28	0.20	0.04	0.14	0.34	1.00
Foreign capital	270	0.89	0.31	0	1	1	1
Location in a big city	270	0.50	0.50	0	0	1	1
Knowledge management adopted (KM)	270	0.30	0.46	0	0	1	1

Note: data for monetary indicators are expressed in million Euros.

**Table 4:** Descriptive statistics of Spanish companies.

Statistic	N	Mean	St. Dev.	Min	Pctl(25)	Pctl(75)	Max
Productivity	1200	0.10	0.42	−2.55	0.01	0.07	7.24
Market value-added (MVA)	1200	336.08	7,268.75	−37,539.06	−112.50	778.00	87,315.91
Economic value-added (EVA)	1200	−317.97	1,210.16	−12,804.16	−175.39	−3.20	5,397.20
Firm's age	1200	50.10	30.40	0	26	71	139
Book value	1200	7,807.67	18,630.15	9.20	292.55	4,183.22	129,775.00
Capital expenses	1200	546.07	3,099.37	−20,256.41	2.08	193.85	41,428.00
Return on invested capital (ROIC)	1200	0.05	0.09	−0.50	0.01	0.07	0.66
Board of directors' qualification (BoDQ)	1200	0.99	0.76	0	0	2	2
Employee cost share	1125	0.20	0.14	0.004	0.11	0.27	1.00
Foreign capital	1200	0.94	0.24	0	1	1	1
Location in big city	1200	0.35	0.48	0	0	1	1
Knowledge management adopted (KM)	1200	0.29	0.45	0	0	1	1

Note: data for monetary indicators are expressed in million Euros.

To examine the descriptive statistics of telecom companies, we collect data from a range of sources, including annual reports, company websites, and industry publications. We analyze the data using a variety of statistical methods, such as means, standard deviations, and percentiles, to identify patterns and trends in the data. However, covering adverse years of economic crisis, the average performance variables - such as productivity, MVA, and EVA - are negative with significant variation. However, ROIC, which is influenced by capital expenses, has a positive average. The companies in the dataset vary in size and age, with some being established in the millennium and others having a century-long history. The human capital of companies is measured by their board of directors' qualifications (BoDQ) and employee cost share. BoDQ is rated on a scale of 0 (low), 1 (medium), or 2 (high) based on the education, professional experience, and tenure of directors in the industry. The heterogeneity of companies also includes the presence of foreign capital, which is measured by a dummy variable in this study. Approximately 30 % of telecom companies and over 94 % of listed Spanish corporations have foreign capital in their financial architecture. Half of the observed companies in both settings are located in large cities. In terms of knowledge management (KM), 46 % of selected telecom companies and 29 % of Spanish companies across all industries have adopted it.

As has been said, one of the key variables we examine in this study is productivity and market value added, which are critical performance indicators of a telecom company's financial health and competitiveness. We examine the performance trends of telecom companies over time, comparing them across all years 2004–2018.

In conclusion, descriptive statistics for Study 1 and Study 2 provides valuable insights into the characteristics of a dataset of telecom companies and large Spanish corporations. By using central tendency, variability, and distribution of the variables of interest we can effectively communicate important features of the data. Further steps in the analysis are required to make meaningful inferences and validate the results of two quasi-experimental studies.

## 44 Empirical results

### 4.1 Study 1. PSM for telecom

As has been mentioned earlier, for Study 1 we employ PSM coupled with OLS estimator to explore the impact of CU on performance in the telecom industry in Europe. We use data from a sample of 18 telecom companies operating in Europe, collected from public sources. We measure CU-driven performance using two objective measures.

(1) productivity to demonstrate whether CU may leverage the return on the human capital of the company, and
(2) MVA as a composite metric of investors' perceptions of CU.

**Fig. 1:**
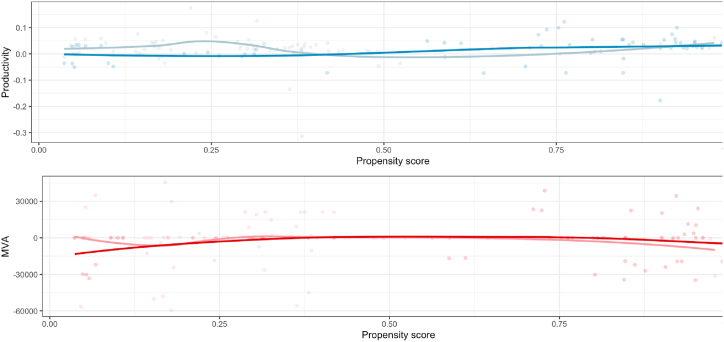
Propensity score matching of telecom companies.

Our study aims to conduct a comprehensive evaluation of the influence of CU on performance, taking into account potential confounding variables that may affect this relationship. We have adopted a two-step approach to achieve this objective. In the first step, we utilized the PSM method (as illustrated in Fig. 1) to generate propensity scores for the observed cases, which consisted of 270 company-year observations. In the second step, we employed the propensity scores to assess whether CU had a significant impact on productivity and MVA while considering the best fit between treated and control companies, as reflected in Table 5. The results obtained from the PSM method demonstrate that the procedure facilitated the maximum possible convergence of treated and control groups. Size of the company, measured by the book value, is not included in the estimation of PSM and will be included as a control in the OLS estimator.

**Table 5:** CU treatment effect for telecom with propensity score matching procedure.

	Dependent variable:	
	Productivity	MVA
	(1)	(2)
CU_treatment	−0.034**	−12,432.970*
	(0.016)	(6,786.998)
Size	0.00000***	−0.083**
	(0.00000)	(0.037)
Distance (PMS)	0.074*	25,748.080*
	(0.038)	(14,968.410)
Constant	−0.109***	−37,163.080**
	(0.041)	(14,765.100)
Observations	132	132
R^2^	0.582	0.602
Residual Std. Error	0.050 (df = 63)	17,810.750 (df = 49)
F Statistic	1.289 (df = 68; 63)	0.905 (df = 82; 49)

*Note:* *p**p***p < 0.01.

However, upon examining the outcomes of our PSM coupled with OLS estimator, we discovered that CU had a negative and significant effect on performance in the telecom industry in Europe. Specifically, the estimated coefficient for CU-driven productivity was −0.034, with a standard error of 0.016, indicating a robust effect. When it comes to CU-driven MVA, the effect was negative but rather noisy, with the estimate being significant at only the 90 % confidence level. This indicates that, on average, CU deflates the market value by 12 million euros, but some companies were still able to generate market value by implementing CU. As a result, we can infer that it is crucial to evaluate CU-driven performance on a case-by-case basis. This finding has prompted us to delve deeper by conducting Study 2, where we will examine one of the observed cases, namely Telefonica CU.

To summarize, our study has presented evidence indicating that CU has a negative average treatment effect on both productivity and MVA within the telecom industry in Europe. These findings remain strong and reliable even when applying various specifications, although there is significant heterogeneity in the observed cases. The results suggest that the formulation and implementation of CU programs should be customized to suit the particular needs and circumstances of each organization, given the heterogeneity of the CU-performance relationship. Therefore, it is crucial to assess different cases of CU separately, and this will be the focus of Study 2.

### 4.2 Study 2. SCG for telefonica

In this study, we have utilized the SCG estimator to investigate the impact of CU on performance within Telefonica, which is one of the most prominent telecom companies in Europe. The main objective of Study 2 is to conduct a rigorous evaluation of the impact of CU on performance, taking into account a counterfactual condition that is constructed using the other telecom corporations and Spanish companies that have never implemented CU.

**Fig. 2:**
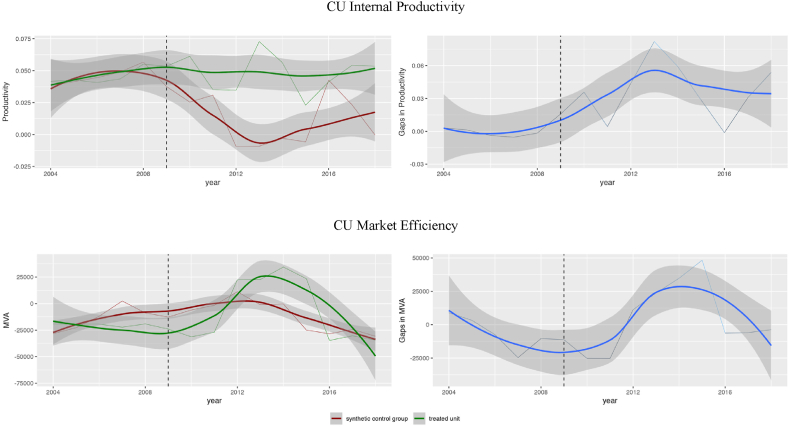
Synthetic control for Telefonica case of CU (synthetic control - telecom companies).

Our analysis, which employs the SCG estimator (as shown in Fig. 2 – top chart), demonstrates that CU has had a positive effect on productivity in Telefonica, starting from the year 2011. This indicates that it took approximately two years for the initial benefits of the CU to materialize in terms of profitability.

**Fig. 3:**
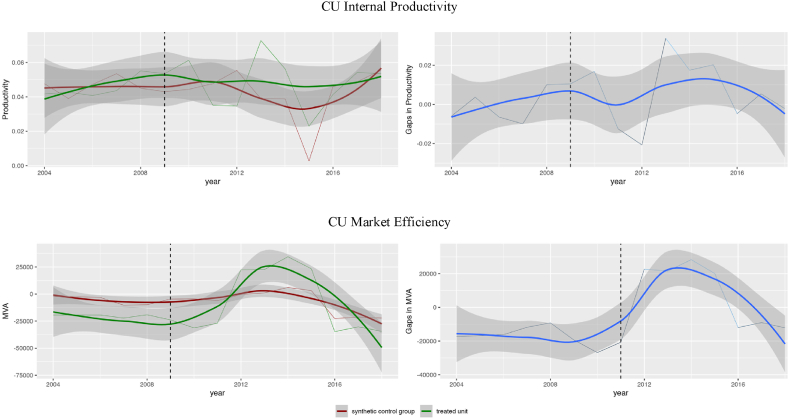
Synthetic control for Telefonica case of CU (synthetic control – all big companies in Spain).

We have estimated that the implementation of CU has led to an average increase in productivity for Telefonica when compared to a synthetic control group of other telecom companies. However, we have not found any significant differences in CU-driven productivity between Telefonica and other large Spanish enterprises (as demonstrated in Fig. 3 – top chart). We have utilized smoothing productivity trends to illustrate whether the confidential intervals intersect or not. The graph on the right side of the figure shows whether the gap between the treated unit and its counterfactual is significantly different from zero. Thus, in the telecom industry as a benchmark, the average increase in productivity after two years of implementing CU varies around 0.04. Which is close to 70 % in relative terms.

The results for MVA as a measure of investors' perception of CU are less sound and delayed. The first positive effect compared to other telecom companies is seen after 2013 and reaches about 15–20 % increase (Fig. 2 - bottom chart). This recognition followed two years of CU-driven productivity gains and four years of overall CU activities. This suggests a lag of about four years before the market started to reflect the positive impact of the CU in the company's valuation.

Additionally, we observed that the period of stable productivity advantages attributable to the CU lasted for at least seven years. During this time, the consistent improvements in productivity underscored the sustained impact of the CU. In terms of investor recognition, this stable period lasted for approximately four years, indicating a continued acknowledgment of the CU's value by the market.

Of note, this effect is also seen as compared to other Spanish companies. However, the effect is tiny and is observed over two years only (Fig. 3 - bottom chart).

To ensure the robustness and validity of our findings, we conducted a placebo test for the SCG estimator. The placebo test involved running the same analysis on a set of placebo-treated units, which are the telecom companies that did not implement CU during the same period as Telefonica. The placebo test allows us to determine whether the positive effect of CU on productivity observed in Telefonica is unique and not spurious. We used the same period and control variables in the placebo test as we did in the analysis of Telefonica.

The results of the placebo test demonstrate that there is no significant effect of CU on productivity for the placebo-treated units, suggesting that the positive effect observed in Telefonica is indeed a result of CU implementation and not due to any other reasons. This provides further support for our main finding that CU has a positive effect both on productivity and MVA in Telefonica with the only expectation when it comes to comparison with the productivity of other Spanish companies.

In conclusion, Study 2 provides evidence that CU can have a positive and significant effect on performance in Telefonica. The results are robust and consistent with the view that CU can be a valuable instrument for improving employee skills, enhancing organizational capabilities, and achieving better business performance. However, the heterogeneity of the CU-performance relationship suggests that the design and implementation of CU are case- and benchmark-specific.

## 5 Discussion of the findings

Our two studies point to a complex relationship between CUs and business performance that varies with context. The first study examined the telecom industry in Europe using PSM combined with OLS estimator and discovered a negative but noisy effect of CU on performance, implying that while CU may have a positive impact on performance in some instances, the effect may not be significant enough to offset those cases where it has a negative impact. The negative average treatment effect of CUs on productivity and MVA within the European telecom industry, as observed in our Study 1, can be explained through a few economic principles and industry-specific factors. Implementing a CU involves substantial investment in terms of both financial resources and managerial attention. In the highly competitive and technology-driven telecom industry, resources diverted to CUs might otherwise be used for critical innovation and immediate operational enhancements. The opportunity costs—what is foregone when resources are allocated to a CU—could potentially outweigh the benefits if the CU does not directly and effectively address the most pressing needs of the company. Of note, if CU programs are not tailored to the specific dynamics and technological demands as evidenced by Refs. [24,31] fail to add value but could also lead to inefficiencies. Generic training programs or those not aligned with the latest technological advancements could render the workforce ill-prepared to meet industry demands, negatively impacting productivity and market valuation. Introducing a CU requires changes in corporate culture and may meet with resistance from employees who are accustomed to traditional ways of working. This resistance can slow down the intended positive impacts of such educational initiatives, leading to reduced productivity in the short term and possibly affecting the long-term strategic positioning of the firm negatively.

On the other hand, the second study focused on Telefonica using SCG and found a strong positive impact of CU on performance when compared to industry counterfactual conditions. This highlights that in specific contexts, CU can be a useful tool for enhancing employee skills, and improving organizational capabilities. Yet, when industry-specific factors are removed from the analysis, these effects are no longer observed. This suggests that the impact of CUs may be contingent upon the distinct features of the CU program and the context of its implementation.

These mixed outcomes are supported by perspective of cases published by Refs. [24,25] suggesting that the impact of CUs on company performance is contingent upon the specific attributes of the CU program and the implementation context. Of note [2,5], and later [2] emphasized the need for CUs to align their educational strategies with the strategic needs of the learning organization for its development.

The findings of Study 2 confirm the contribution by Ref. [1] who assert that the implications for internal productivity are particularly noteworthy. The mechanism by which CUs contribute to productivity was not explicitly examined in our studies; however, it is hypothesized along with previous findings of [10,31] that CUs have the potential to bolster productivity through the enhancement of employee skills and knowledge, the cultivation of a learning culture, and the promotion of collaboration. Collectively, these elements can lead to significant improvements in a company's internal dynamics, resulting in cost reductions, accelerated innovation, and improved product quality—all of which are likely to have a positive impact on a firm's overall success. This assertion aligns with the findings from Telefonica's productivity gains are clearly evident when compared to its direct competitors in the telecom industry. While Telefonica's productivity improvements reflect the potential benefits of a well-implemented CU, the expected positive impact on MVA was less pronounced. This outcome could indicate investors' hesitance to fully value the impact of CUs on a company's market value, despite observable operational advancements. This supports previous research by Refs. [4,41], which suggest a weak link between CUs and market value created. According to Ref. [42], CU may be a subject of a corporate agent conflict resulting in a more limited impact on market value than on firm productivity, as market value reflects not only the firm's internal resources and capabilities but also subjective perceptions of investors beyond the firm's management control.

Furthermore, Study 2 presents evidence suggesting that the relationship between a CU and performance can differ based on the benchmark used for comparison. The beneficial impact of Telefonica's CU is observed only when comparing it to other European telecom companies that do not have CUs to synthesize a control group. However, when Telefonica is compared to a broader pool of large Spanish corporations across various industries, the CU's effect is no longer discernible, showing neither positive nor negative outcomes. While CUs have been shown to enhance productivity by developing employee skills and fostering a culture of learning, the extent of these benefits can be industry-specific, providing advantages that are context-dependent. Studies by Refs. [8,22] indicate that the gains from CUs, such as cost savings, accelerated innovation, and improved product quality, are particularly pronounced in sectors where these factors align closely with industry demands. Therefore, the impact of CU programs on internal productivity is most significant when they are tailored to the unique requirements of the job and the industry, emphasizing skills such as leadership, communication, and problem-solving.

Moreover, the positive effects of CUs on market value may also be circumscribed by industry characteristics. As demonstrated by Refs. [4,30], CUs that invest in strategic capabilities and signal a commitment to innovation can influence market valuation favourably. However, this influence is most potent within industries where innovation and strategic adaptation are key drivers of growth. Hence, the advantage of CUs in enhancing market value might not be universally applicable across sectors, especially when comparing companies from different industries where the strategic priorities and investor expectations may vary significantly.

### 5.1 Theoretical and managerial implications

Our findings contribute to the theoretical understanding of CUs a strategic choice of a firm by highlighting the conditional nature of their impact on corporate performance.

They challenge the traditional view that CU benefits are universally applicable, highlighting that the positive outcomes of CUs are sensitive to industry norms and the specific competitive landscape. This emphasizes the importance of viewing CUs as a strategic choice that requires careful consideration and customization to align with the unique demands of each industry. The findings have significant implications for theories of strategic human capital development, indicating that a tailored approach is essential when integrating CUs within a firm's broader strategic framework.

The results also support the theory that the alignment of educational strategies with business objectives is crucial for the successful application of corporate learning initiatives. By strategically aligning CUs with the company's goals and competitive environment, firms can maximize the impact of their educational investments, ensuring that their workforce development efforts directly contribute to achieving strategic objectives. This strategic alignment underscores the role of CUs as vital components of a company's overall strategy, rather than standalone educational entities.

Considering that for the majority of CUs, as our analysis in Study 1 demonstrates, can be too costly and do not bring positive performance responses on average, it is crucial to approach CU implementation with a specific corporate strategy. However, there are instances, as shown in Study 2, where CUs prove to be reasonable investments. Therefore, policy implications should be chosen accordingly.

It is essential to go beyond a mean analysis and instead focus on identifying relevant and successful cases to use as benchmarks. Each company should carefully evaluate these benchmarks, understanding the specific conditions and factors that led to their success. Additionally, it is important to assess all potential risks and deviations from the benchmark outcomes when implementing this strategic decision for each particular company. This tailored approach will help ensure that CUs are a strategic choice that aligns with the unique needs and circumstances of the organization, maximizing the chances of positive performance outcomes.

Our findings have implications for companies contemplating investing in employee education and training through CU programs. While CU can have a positive impact on performance, it is not a one-size-fits-all solution. The design and implementation of CU programs should be customized to the specific needs and context of the organization to ensure maximum effectiveness. To achieve this, companies should carefully evaluate the features of the CU program, such as the level of employee participation, the type of training, and the geographic location, to ensure that the program aligns with the company's goals and objectives.

Thus, for managers and policy makers, the studies underscore the importance of context when designing and implementing CU programs. Our research suggests that CUs should not be deployed as a blanket strategy across an organization, but rather be tailored to address the specific strategic needs of different divisions or sectors within a firm. Managers should also be mindful of industry-specific benchmarks when evaluating the success of CUs, recognizing that enhancements in productivity or market value may not be directly transferrable to different industries or competitive environments.

Furthermore, the differential impact of CUs on productivity versus market value suggests that while CUs can be instrumental in driving internal improvements, their influence on external stakeholders' perceptions may require additional communication and engagement strategies. Managers may need to proactively showcase the value generated by CUs to investors and other external parties to realize their full benefit on market valuation.

To effectively leverage the advantages of CUs as a strategic choice, managers are encouraged to:

(1) Conduct thorough industry-specific analyses and relevant case-study comparison to determine how CUs can best contribute to strategic goals of their firm. This ensures that CU initiatives are tailored to the unique competitive landscape and industry norms, maximizing a probability of their positive impact on the firm's performance.
(2) Communicate the value proposition of CUs to external stakeholders, including investors, to bridge the gap between internal improvements and market valuation. Highlighting the strategic role of CUs can enhance investor confidence and support.
(3) Develop CU curricula that are closely aligned not only with the current but future skill requirements of the firm. By doing so, CUs must be seen a long-term perspective supporting the future company's strategic objectives and prepare the workforce for emerging challenges and opportunities.
(4) Continuously monitor and adapt CU programs in response to evolving industry demands and strategic shifts within the firm. This ensures that CUs remain relevant and effective as strategic tools for achieving long-term business success.
(5) Integrate CU initiatives with broader corporate strategies, ensuring that CUs are not standalone entities but are fully embedded within the company's strategic planning. This integration helps create a cohesive approach to human capital development that supports overall business objectives and drives sustainable competitive advantage.

By understanding the conditional effects of CUs demonstrated in our studies, managers and organizations can better position their CU strategies to enhance both internal productivity and external valuation, contributing to sustained competitive advantage.

### 5.2 Limitations and future research avenue

It is important to acknowledge the limitations of our studies. The sample size of both studies is relatively small, which may limit the generalizability of our findings to other industries and contexts. Additionally, the data we used was observational, which may introduce selection bias and omitted variable bias, potentially skewing the relationship between CUs and performance. While the methodology of this study is rigorously detailed and offers a comprehensive approach to evaluating the impact of CUs on corporate performance, it is important to note potential limitations regarding its replicability. The applicability of our quasi-experimental design relies on the availability of extensive longitudinal data and a specific context that may not be readily available or applicable in other sectors. The data preparation and the particular settings required for the PSM and SCG methods mean that replication of the study demands a similarly detailed dataset and careful consideration of contextual factors.

Another significant limitation of our study is the lack of investigation into the specific mechanisms by which CUs translate into improved performance. Understanding the direct pathways through which CUs influence various performance measures remains an area that was not addressed in our research.

Despite these limitations, we have made efforts to mitigate potential biases through robust methodological approaches, including the use of placebo tests. Our findings align with established theoretical frameworks and corroborate prior empirical research, suggesting they hold relevance despite the constraints.

Looking forward, several paths for future research could expand our understanding of CUs and their impact on performance. Future studies could:

(1) Increase sample sizes and include a broader range of industries to enhance the generalizability of findings.
(2) Replicate longitudinal data to examine the long-term effects of CUs on performance based on specific cases where the SCG method can be taken as an effective empirical strategy.
(3) Investigate the specific features of CU programs that correlate with the most significant performance improvements.
(4) Explore the role of industry-specific dynamics in shaping the effectiveness of CUs.
(5) Examine the potential moderating effects of organizational culture and leadership on the CU-performance relationship.
(6) Consider the impact of digital and remote learning platforms as part of CU strategies, especially in response to recent global shifts toward virtual work environments.
(7) Study the relationship between external perceptions of CUs and actual performance outcomes, particularly in relation to market value.

### 5.3 Conclusions

In conclusion, our study contributes to the academic and practical understanding of the relationship between CU and performance. Our findings suggest that while CU can have a positive impact on performance, the effect may vary depending on the specific features of the program and the context in which it is implemented. Future research should explore the mechanisms through which CU affects performance and identify the specific features of the program that are most effective in enhancing employee skills and organizational capabilities. By doing so, companies can make informed decisions about designing and implementing CU programs that will have a positive impact on their business performance.

## Ethics statement

Not applicable.

## Data availability statement

Data will be made available on request.

## Ethics and consent statement

The current study does not include human or animal participation.

## CRediT authorship contribution statement

**Elena Shakina:** Writing – original draft, Visualization, Validation, Software, Methodology, Investigation, Formal analysis, Data curation, Conceptualization. **Ángel Barajas:** Writing – review & editing, Validation, Supervision, Investigation, Formal analysis, Conceptualization. **Patricio Sánchez-Fernández:** Writing – review & editing, Resources, Project administration, Investigation, Funding acquisition.

## Declaration of competing interest

The authors declare the following financial interests/personal relationships which may be considered as potential competing interests:Elena Shakina reports financial support was provided by 10.13039/501100007251National Research University Higher School of Economics. Patricio Sanchez-Fernandez reports financial support was provided by 10.13039/501100010801Xunta de Galicia. If there are other authors, they declare that they have no known competing financial interests or personal relationships that could have appeared to influence the work reported in this paper.

## Footnotes

2 Productivity is measured as value-added per employee.

3 BoDQ is rated on a scale of 0 (low), 1 (medium), or 2 (high) based on the education, professional experience, and tenure of directors in the industry.

4 Percentage of total costs that are attributed to employee-related expenses.

5 A dummy variable that takes the value of 1 when there is foreign capital in the company's capital and 0 otherwise.

6 A dummy variable that takes the value of 1 when the CU is located in a city with more than 1 million inhabitants, and 0 otherwise.

7 A dummy variable that takes the value of 1 when the knowledge management (KM) system has been adopted by the company and 0 otherwise. This is the result of a search on company location on their website using the following words as “knowledge management”.

## Footnotes

1 https://www.archdaily.com/214107/telefonica-corporate-university-in-parc-de-bell-llo-percent-e2-percent-80-percent-8b-batlle-roig-architects.
